# RGB color correction and gamut limitations in smartphone-based kinetic analysis of chemical reactions

**DOI:** 10.1007/s00216-025-06021-9

**Published:** 2025-08-15

**Authors:** Calum Fyfe, Shengkai Yu, Jing Zhang, Marc Reid

**Affiliations:** 1 Department of Pure and Applied Chemistry, University of Strathclyde, Royal College Building, 204 George St., Glasgow, G1 1BX UK; 2 National Metrology Center (NMC), A*STAR, 8 Cleantech Loop, Singapore, 637145 Singapore

**Keywords:** Digital image colorimetry, Video analysis, Smartphone colorimetry, Color correction, Computer vision

## Abstract

**Abstract:**

The variability in hardware specifications and environmental factors poses significant challenges to the use of smartphone cameras in analytical measurement. Towards time-resolved color analysis and reaction monitoring, we systematically quantified multiple sources of measurement uncertainty in smartphone-based color measurements, finding that while sensor repeatability is high ($\Delta E < 0.5$), lighting conditions and viewing angles can introduce substantial bias ($\Delta E$ versus reference colors increasing by up to 64% at oblique angles). We implemented and evaluated a matrix-based image color correction methodology using a color reference chart, reducing inter-device and lighting-dependent variations by 65–70% (quantified by the color change metric, $\Delta E$). Moving beyond static image correction to video analysis, our approach was validated through the monitoring of Blue1 dye degradation kinetics using videos recorded on two different smartphones. Time-resolved and color-corrected measurements from both devices produced consistent kinetic profiles. Importantly, we identified a fundamental limitation in RGB-based colorimetry: highly saturated colors that exceed the sRGB color gamut create artificial discontinuities in kinetic profiles, manifesting as “shouldering” effects not present in spectrophotometric data. Unlike previous methods that focused on controlling environmental factors through custom enclosures, our time-resolved color correction methodology systematically quantifies and corrects for multiple sources of color bias across various smartphone models, enabling standardized measurements even in variable conditions. This advancement enhances the reliability of field-ready, smartphone-based colorimetric applications and establishes a framework for calibrating video-based reaction monitoring against established spectroscopic measurements.

**Graphical abstract:**

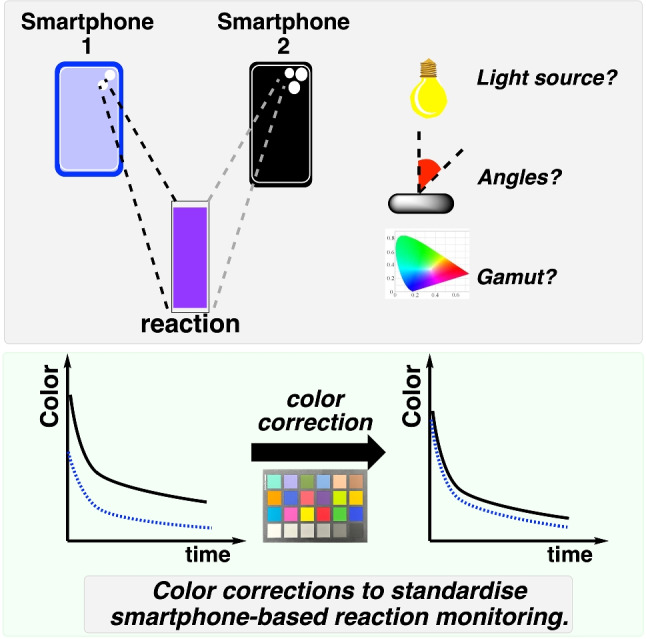

## Introduction

**Fig. 1:**
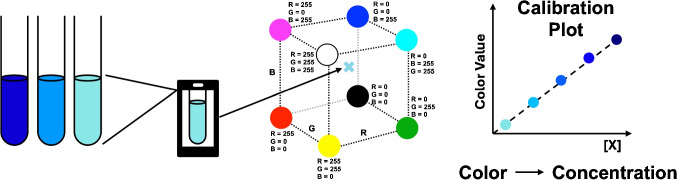
Digital image colorimetry (DIC). A smartphone camera (or any device capable of capturing a digital image) is used to capture an image of the colored sample. The color values are then extracted from the image, typically expressed as RGB values. The colors from known samples are used to generate a calibration plot, which can then be used to determine the concentration of an unknown sample based on the color obtained from a smartphone image

Digital image colorimetry (DIC) is a non-contact technique that quantifies an analyte’s concentration using color values extracted from a digital image (Fig. 1).

**Fig. 2:**
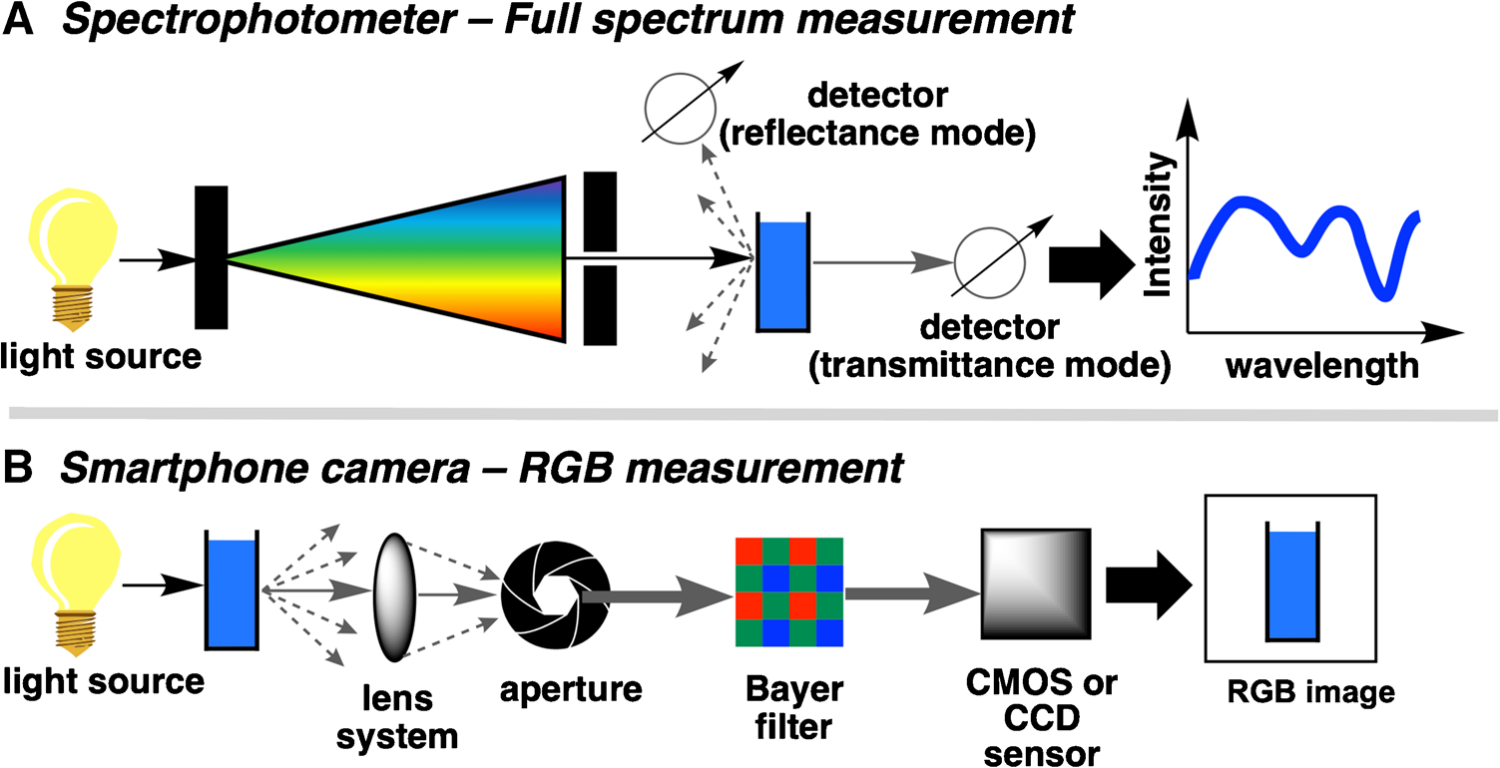
Comparison of color measurement approaches. **A** Spectrophotometer measures the complete visible spectrum of transmitted or reflected light (top). **B** A smartphone camera captures color using a Bayer filter array that measures three broad color channels (bottom)

While applications of DIC date back to the early 2000s [1], publication activity has since surged with the ubiquity of high-quality smartphone cameras [2]. Recent applications [3, 4] demonstrate the versatility of this approach across diverse analytical challenges: detecting disease markers in blood, [5] proline detection in food samples, [6], and ammonium and nitrate in soil samples [7]. In education, the accessibility of smartphone DIC was leveraged during the COVID-19 pandemic to enable remote practical lessons of analytical techniques [8]. There are also cases of using machine learning and artificial intelligence in DIC [9–11]. For example, Cánovas-Saura and co-workers have employed neural network-assisted procedures for visible spectrum reconstruction, using only the camera-extracted color values. Such methods have the potential to positively impact the accuracy of color measurements over large surface areas [12].

Despite its evident applicability, DIC faces a fundamental challenge, namely the reproducibility of color values captured by different smartphone cameras used under varying conditions [3]. Unlike traditional spectrophotometers, a smartphone camera does not measure a spectrum. They capture an image by directing light through a lens system and onto an array of photosensitive sites on a semiconductor, typically covered with a Bayer color filter. A simplified overview of spectrophotometer and camera measurement methods is shown in Fig. 2.

The variability of smartphone color measurement stems from two key sources: hardware diversity (e.g., phone brand + model) and capture conditions (i.e., lighting, angle, and other environmental factors). Smartphone cameras utilize different sensors, lenses, and processing algorithms that, together, affect the quantified color output [13]. Ambient lighting conditions significantly impact color values, as smartphone cameras lack the controlled light paths of laboratory spectrophotometers.

Most current solutions to color measurement involve controlling the measurement environment through custom-desig-ned enclosures and standardized lighting [14–16]. A promising but underexplored approach to more robust DIC is image color correction. Image color correction is widely used in photography and digital imaging, but remains underutilized in digital analytical chemistry. Image color corrections can make DIC robust to different lighting conditions and camera sensors. Cao and Jai et al. have shown the use of image color correction to reduce external interference in smartphone DIC for paper-based colorimetric tests [17]. Color correction was also important for machine learning training [12].

Recent work by Cebrián et al. demonstrated a comprehensive approach to smartphone color standardization using RAL Classic color charts and 3D-printed light boxes, achieving significant reduction in measurement variability between devices for static colorimetric analysis [18]. While their methodology successfully addressed device-to-device variations for single-point measurements using linear least-squares correction matrices, it focused primarily on controlled laboratory conditions with fixed lighting environments. Our approach extends beyond static image correction to address the unique challenges of dynamic video-based reaction monitoring, where temporal color changes must be tracked continuously across varying environmental conditions. Unlike previous methods that rely on controlled enclosures to minimize external interference, our color correction methodology systematically quantifies and corrects for multiple sources of color bias (versus reference colors) across various smartphone models, enabling standardized measurements even in variable field conditions. Furthermore, we identify and characterize a fundamental limitation in RGB-based colorimetry that has not been previously documented: the artificial discontinuities created when highly saturated colors exceed the sRGB color gamut during kinetic monitoring, manifesting as “shouldering” effects that do not appear in spectrophotometric data.

### Study aims

Our team has developed *Kineticolor*, a computer vision software that enables video and image analysis for non-contact kinetic analysis of chemical reaction bulk. The technology has been applied, for example, to mixing analysis for reaction scale-up [19], high throughput kinetics and discovery [20], enhanced forensic spot tests [21, 22], and transition metal catalyst degradation [23]. While our applications of computer vision reaction monitoring have been varied and useful, we had not, until the present study, applied any color correction methods to raw data during analysis.

By enabling higher agreement of color measurements collected on different cameras trained on the same chemical process, we envisaged unlocking new opportunities for multi-camera process monitoring that are currently beyond the scope of our capabilities. Motivated by this goal, this study addresses three key challenges in smartphone-based digital image colorimetry:

1. Quantifying the major sources of bias (i.e., deviation from reference colors) in smartphone color measurements, including sensor, angle, and lighting variations
2. Developing and evaluating an image color correction methodology that enables agreement of color measurement across different smartphone cameras and environmental conditions
3. Demonstrating the application of this approach to time-resolved monitoring of Blue1 dye degradation by sodium percarbonate, establishing the viability of digital video colorimetry for reaction kinetics studies

Our results provide a practical framework for improving the reproducibility and reliability of smartphone-based video data for the purpose of time-resolved non-contact analysis of chemical and biochemical reaction bulk, as a complement to the broader suite of molecularly specific process analytics.

## Methods

### Spectra to color

To capture reference colors against which to measure bias of color measurements taken on the smartphones, a PerkinElmer LAMBDA 1050+ UV/Vis/NIR Spectrophotometer was used to capture the visible region transmission and reflectance spectra of all 24 color patches on the Datacolor Spyder Color Checkr and from cuvettes containing reacting Blue1 samples (see the “Reaction monitoring experiments” section). From the visible region, $\sim$400–700 nm, transmission or reflectance spectra were converted to color values. Formally, conversions involved integrating the functions of the spectral data ($R(\lambda )$) with a standard illuminant($S(\lambda )$) and standard observer functions ($\bar{x}(\lambda ), \bar{y}(\lambda ), \bar{z}(\lambda )$). However, as the spectral data are measured at discrete wavelengths, the computation is based on the summation of these values. The summation is shown in Eq. 1, which provides CIE *XYZ* color values. The CIE *XYZ* color values were calculated using both the CIE D65 and D50 standard illuminants and the CIE 1931 2$^{\circ }$ standard observer functions. These standard functions were obtained from the Commission Internationale de l’Éclairage (CIE) database at 1 nm resolution between 360 and 830 nm. In the machine-readable supporting information (https://doi.org/10.6084/m9.figshare.28996382.v3), the D65, D50, and color chart manufacturer reference values for the 24-color chart are made available in spreadsheet form.

1 $$\begin{aligned} X= &   \frac{\sum _{\lambda }R(\lambda )S(\lambda )\bar{x}(\lambda )}{\sum _{\lambda }S(\lambda )\bar{y}(\lambda )}\quad Y=\frac{\sum _{\lambda }R(\lambda )S(\lambda )\bar{y}(\lambda )}{\sum _{\lambda }S(\lambda )\bar{y}(\lambda )}\nonumber \\ Z= &   \frac{\sum _{\lambda }R(\lambda )S(\lambda )\bar{z}(\lambda )}{\sum _{\lambda }S(\lambda )\bar{y}(\lambda )} \end{aligned}$$

### Color conversions

The CIE *XYZ* color space is the foundational color space of the CIE system. The CIE *XYZ* values can be converted to other color spaces. CIE *xyY* is another useful color space. It is common to see this information as an *xy* chromaticity diagram to compare the color of samples independent of illuminance (*Y*).

2 $$\begin{aligned} x=\frac{X}{X+Y+Z}\quad y=\frac{Y}{X+Y+Z}\quad Y=Y \end{aligned}$$

CIE $L^*$ $a^*$ $b^*$ is another useful color space. It is intended to be a perceptually uniform color space. It is based on the opponent model of human vision, where red and green form an opponent pair, and blue and yellow another. The relationship between the $L^*$, $a^*$, and $b^*$ channels to the *XYZ* color space are shown in Eqs. 3–5. This color space is beneficial for comparing colors via the color-agnostic color difference (or contrast) metric, $\Delta E$, which is defined later in the paper.

3 $$\begin{aligned} L^*= &   116^* f \left( \frac{Y}{Y_n}\right) +16,\end{aligned}$$

4 $$\begin{aligned} a^*= &   500\left( f\left( \frac{X}{X_n}\right) -f\left( \frac{Y}{Y_n}\right) \right) ,\end{aligned}$$

5 $$\begin{aligned} b^*= &   200\left( f\left( \frac{Y}{Y_n}\right) -f\left( \frac{Z}{Z_n}\right) \right) \end{aligned}$$

where *t* is $$\frac{X}{X_n}, \frac{Y}{Y_n}, or \frac{Z}{Z_n}\quad f(t)={\left\{ \begin{array}{ll} \root 3 \of {t}&  \text {if }t>\frac{6}{29}^3\\ \frac{841}{108}t+\frac{4}{29} &  \text {otherwise} \end{array}\right. }$$

$$\begin{aligned} \begin{array}{lll} \text {For D65} &  \text {For D50}\\ X_n=95.0489, &  X_n=96.4212,\\ Y_n=100, &  Y_n=100,\\ Z_n=108.8840 &  Z_n=82.5188 \end{array} \end{aligned}$$

The CIE *XYZ* values can also be transformed into variations of the RGB color space. The most common variant is D65 sRGB color space. CIE *XYZ* is converted to D65 sRGB through linear RGB D65.

6 $$\begin{aligned}  &   \text {CIE XYZ to linear rgb D65} \begin{bmatrix} r \\ g \\ b \end{bmatrix}= \begin{bmatrix} 3.2405 &  -1.5371 &  -0.4985 \\ -0.9693 &  1.8760 &  0.041556 \\ 0.0556 &  -0.2040 &  1.0572 \end{bmatrix} \begin{bmatrix} X \\ Y \\ Z \end{bmatrix}\end{aligned}$$

7 $$\begin{aligned}  &   \text {linear rgb D65 to D65 sRGB}\quad \begin{matrix} V={\left\{ \begin{array}{ll} 12.92v&  \text {if }v \le 0.0031308\\ 1.055v^{\frac{1}{2.4}}-0.055 &  \text {otherwise} \end{array}\right. } \end{matrix} \\  &   \textit{v} \in {r,g,b} \nonumber \\  &   \textit{V} \in {R, G, B}\nonumber \end{aligned}$$

### Color difference measurements

In all cases where the contrast or difference between two color or light samples was required, the suite of $\Delta E$ definitions, derived from CIE–$L^*$ $a^*$ $b^*$ color measurements, were applied. All $\Delta E$ definitions are available in the $\Delta E$ measurement script available in the supporting information, including $\Delta E_{94}$, $\Delta E_{2000}$, and $\Delta E_{CMC}$.

For a straightforward demonstration of improvement (or not) in color agreement after correction, the simplest definition, $\Delta E_{76}$, was applied throughout this manuscript. It is defined by the Euclidean (straight line) distance between two colors across $L^*$ $a^*$ $b^*$ space.

8 $$\begin{aligned} \Delta E_{76} = \sqrt{(L_2^* - L_1^*)^2 + (a_2^* - a_1^*)^2 + (b_2^* - b_1^*)^2} \end{aligned}$$

where $L_1^*, a_1^*,$ and $b_1^*$ are the CIELAB coordinates of the reference color and $L_2^*, a_2^*,$ and $b_2^*$ are the CIELAB coordinates of the sample color. See also Fig. 5.

### Correction methods

#### Reference standards for reflectance spectra correction

A reflectance reference standard with a known reflectance, traceable to national or international standards, was used to correct the reflectance spectra of color samples. We used Opal JK97 as the white reflectance standard (WS). Reflectance spectra, $S_R$, were corrected with reference to this standard, correcting for the difference between the known reflectance of the reference standard $WS_{known}$ and the reflectance of the sample, measured by the PerkinElmer LAMBDA 1050+ spectrophotometer, $WS_R$.

9 $$\begin{aligned} S_{R_{corrected}}=S_R\cdot \frac{WS_{known}}{WS_R} \end{aligned}$$

#### Color correction

Color correction involves transforming the RGB values of an image to match a set of reference color values measured on an independent instrument. This transformation can be represented as a linear mapping using a $3 \times 3$ correction matrix, *M*. Reference color values can be taken from a manufacturer or from experiment (e.g., from reflectance spectra). Commercially available color charts, such as the Datacolor Spyder Checkr 24, provide a $6\times 4$ card of known color values: aqua, lavender, evergreen, steel blue, classic light skin, classic dark skin, primary orange, blueprint, pink, violet, apple green, sunflower primary cyan, primary magenta, primary yellow, primary red, primary green, primary blue, card white, 20 gray, 40 gray, 60 gray, 80 gray, and card black. The RGB values for these 24 colors can be used as reference colors for camera image color correction. With the Datacolor Spyder Checkr 24 made visible within the camera’s field of view, it enables color correction of the whole image (or series of video frames) recorded on the camera. With both the color chart references colors—$R_{ref(n)}$, $G_{ref(n)}$, and $B_{ref(n)}$—as determined from the spectra, and the corresponding colors extracted from the image/video frame, then a 3$\times$3 correction matrix, *M*, can be determined. Determining *M* was approached programmatically in Python using a least-squares optimization approach implemented in NumPy (numpy.linalg.lstsq). This method minimizes the sum of squared differences between the reference colors and the measured colors, determining *M* to minimize the difference between the reference color values and those captured in the image or video frame (Eq. 10). Once *M* has been determined, it can be applied to all pixels in an image to correct the color in the whole image or video frame.

10 $$\begin{aligned} \begin{bmatrix} R_{ref1} &  G_{ref1} &  B_{ref1}\\ R_{ref2} &  G_{ref2} &  B_{ref2}\\ \vdots &  \vdots &  \vdots \end{bmatrix}= M \times \begin{bmatrix} R_{1} &  G_{1} &  B_{1}\\ R_{2} &  G_{2} &  B_{2}\\ \vdots &  \vdots &  \vdots \end{bmatrix} \end{aligned}$$

### Reaction monitoring experiments

#### Blue1 dye stock solution

A 10 mM stock solution of Blue1 (1 mmol, 0.792 g) was prepared in a 100-mL volumetric flask. The 10 mM stock was used to prepare all the Blue1 calibration samples and Blue1 samples for the degradation experiment.

#### Calibration samples

Calibration samples (3 mL each) were prepared in standard 1-cm path length cuvettes from the 10 mM stock solution of Blue1 through a series of dilutions, producing calibration solutions of 5, 3, 1, 0.5, 0.3, 0.1, 0.05, 0.03, 0.01, 0.005, 0.003, 0.001, 0.0005, 0.0003, and 0.0001 mM.

**Fig. 3:**
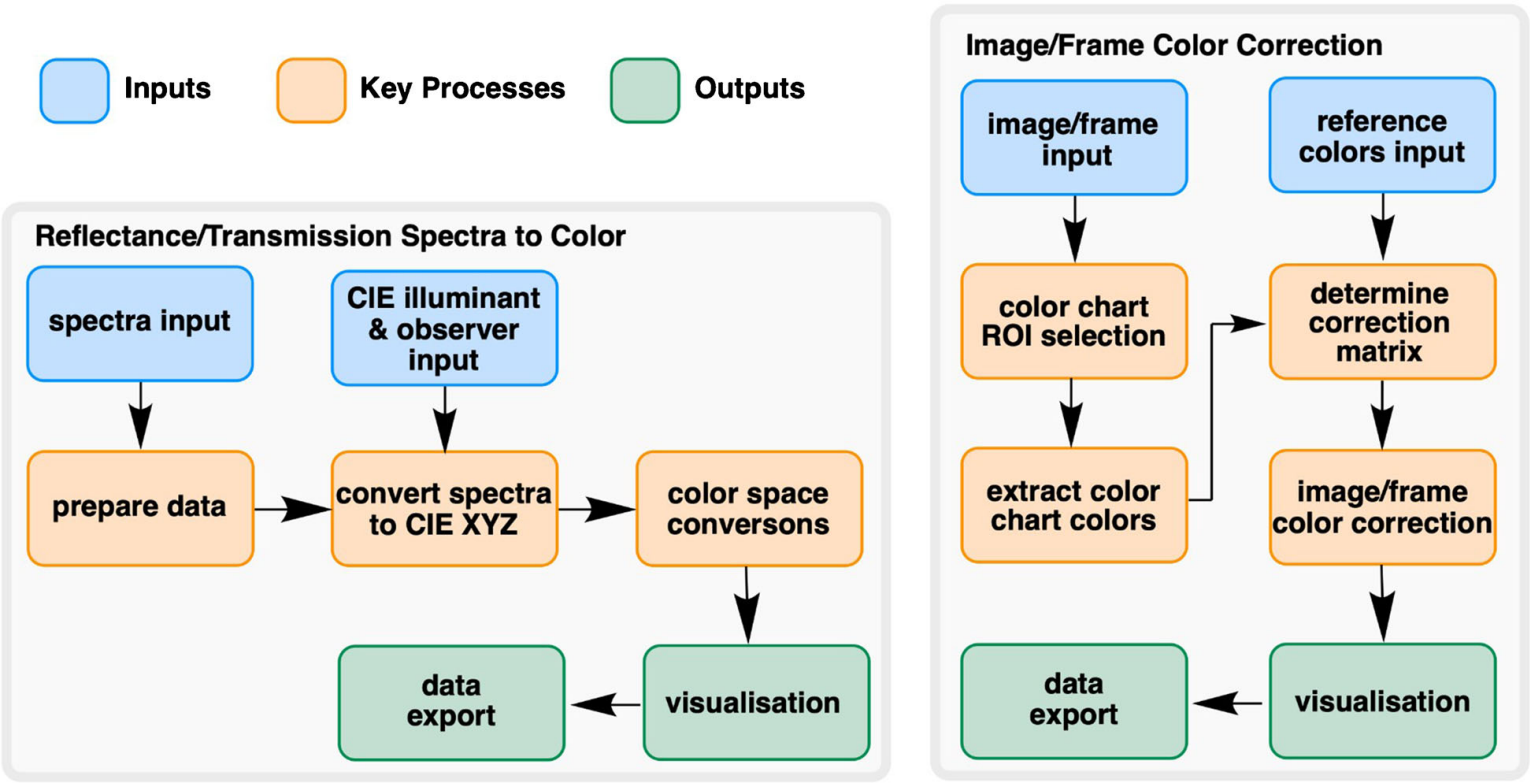
Overview of the analytical workflow developed for color correction and calibration in video-based reaction monitoring. The diagram illustrates the relationship between key steps for image extraction, region-of-interest (ROI) selection, color calibration, and time-resolved analysis. The workflow enables standardization of color monitoring measurements across different cameras and lighting conditions

#### Degradation experiments

All degradation experiments were conducted at room temperature. 0.3 mL of the 10 mM stock solution of Blue1 and 2.6 mL of water was added to a cuvette (path length = 1 cm). Next, 3 mL of a 0.3 M sodium percarbonate (0.141 g) working solution was prepared. The sodium percarbonate solution was only prepared as and when needed as the percarbonate solution decomposes in aqueous solution. Once prepared, 0.1 mL of the sodium percarbonate working solution was transferred to the cuvette containing Blue1, using a micropipette to begin the dye degradation experiment. The resultant solution was mixed by repeatedly dispensing and withdrawing the solution with the micropipette. After mixing, the reaction progress was followed either by a spectrophotometer or by a video captured using one of two smartphone models employed in this study.

### Imaging methods

Two smartphone cameras were used to capture images and video:

1. **iPhone 14 Pro** (48 MP, f/1.8 aperture, 24 mm equivalent focal length main camera, sensor sony IMX803 quad-Bayer CMOS sensor. The 3rd party camera app Halide Mark II Pro was used to enable more control over the camera settings when capturing images. Videos were captured using the native iOS Camera app.
2. **Samsung S23** (50 MP, f/1.8 aperture, 23 mm equivalent focal length, main camera, Samsung ISOCELL GN5 CMOS sensor). The 3rd party Open Camera app was used for the capture of both images and videos.

An indicator transfer stand and bike smartphone holder were used to secure the smartphone during image or video capture, ensuring a steady, unmoving field of view was recorded in the images and video frames. The level functionality in the iPhone’s native Measure app was used to control the capture angle. In relation, the distance between the camera lens and the sample was maintained.

### Code

To support core spreadsheet-based analysis, a collection of Python scripts were developed to enable and automate spectral and image/video data processing. The main analytical workflow is illustrated in Fig. 3. The Python scripts, documentation, and example inputs (where appropriate) are all provided in the supporting information.

#### Spectra to color

Wavelengths and intensity responses were extracted from the spectrophotometer output .acs file, removing unnecessary data for the color calculation. With the transmittance or reflectance data provided, the Python script used to handle the spectrum to color conversion can also handle corrections to white and black reflectance standards. With the spectral data, corrected or not, the CIE Standard illuminants and observer functions are filtered based on the sample wavelengths measured. The *XYZ* values were then calculated and translated into other color spaces, such as CIE *xyY*, CIE $L^*$ $a^*$ $b^*$, and sRGB.

#### Image analysis and comparison

Image analysis involved loading an image and selecting a ROI. RGB summary statistics (mean, mode, median, etc.) were determined from all the pixels included in the ROI. The mean RGB color values were then used for conversion to the other color spaces. Two images can be loaded for comparison, and the user can select an ROI on each image. The script then calculates the average RGB values of the pixels in each ROI and converts them to CIE $L^*$ $a^*$ $b^*$ to enable color difference between the ROIs to be calculated via $\Delta E$.

#### Image color correction

Reference color values are preloaded in the program from a JSON file containing the spectra-determined color values of a Datacolor Sypder Checkr 24. First, the image to be color-corrected is loaded. The script then enables the user to position a 6 x 4 grid over the Datacolor Spyder Checkr 24 in the image. Once positioned, the image is ready for correction. The average RGB color value is extracted from the pixels from each grid square. Now, with the reference values of the squares and those from the images, the 3$\times$3 correction matrix is determined using the NumPy linalg.lstsq method, a least squares approach for matrices. This method returns the matrix that, when multiplied with the image square color values, returns values that match the reference colors or at least a closer match. With *M* determined, the image can be color-corrected by applying *M* to all pixels. For comparison, the script calculates $\Delta E$ of the color chart colors before and after correction compared to the reference value. A batch version was developed to take a folder of images as an input, intended for color correction of extracted video frames, where the color chart does not move such that the same grid positioning works for all images.

#### Video analysis

The first step was to load a video and extract the frames based on a user-defined frame sampling rate. Each frame was cropped to a user-selected ROI. One ROI was set and applied to cropping of all subsequent frames. The extracted frames were saved individually with names based on the time each frame appeared in the video. Batch image color correction was applied to the folder of extracted frames. Like the image analysis, the average color values were determined from within the ROI of each extracted video frame in the folder, producing time series data based on averaged color.

**Fig. 4:**
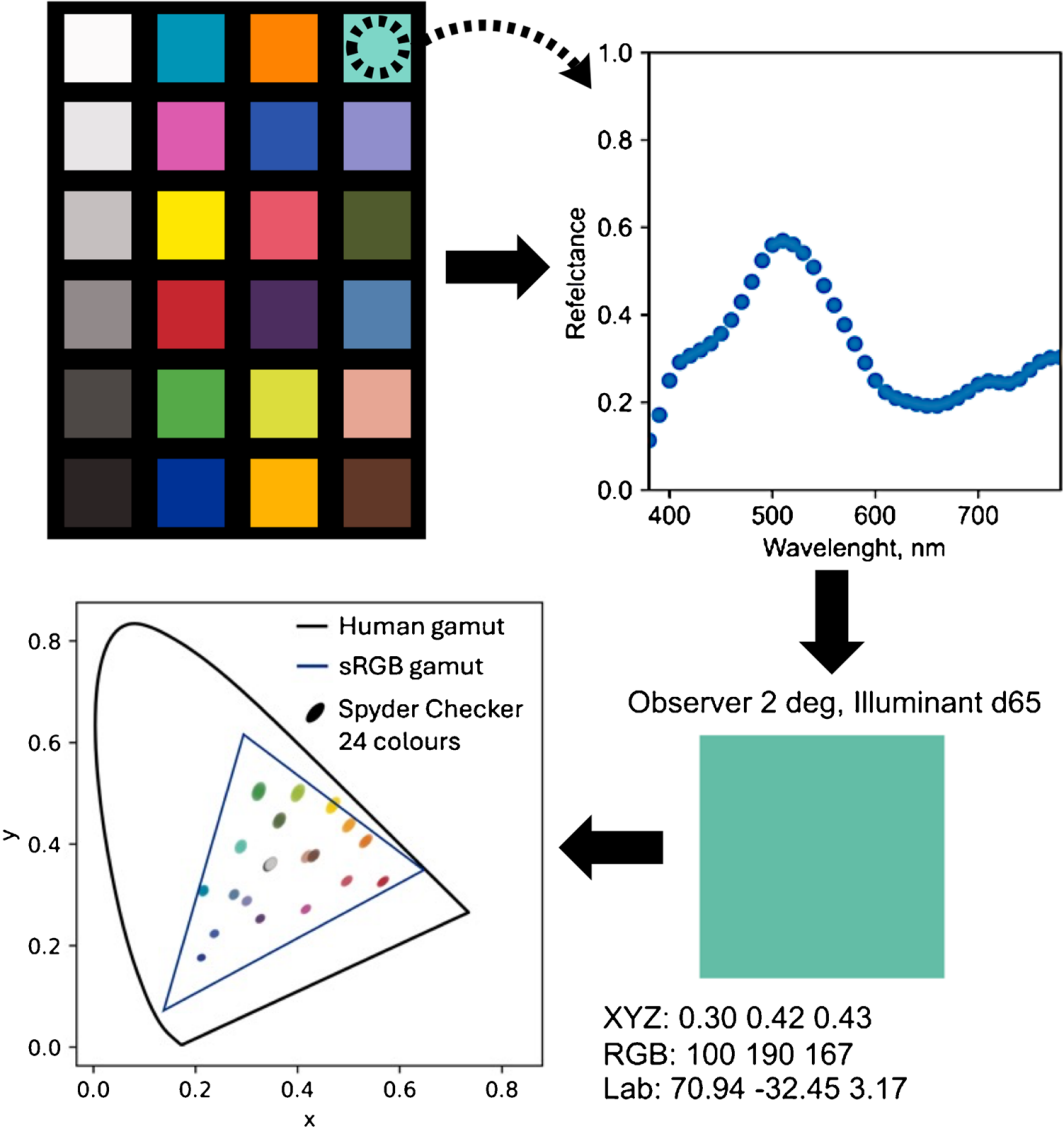
Characterization of reference color standards. Top left: the Datacolor Spyder Checkr 24 color chart used for color correction. Top right: measured reflectance spectrum for the Aqua color patch (top right patch on the color card). Bottom right: color values calculated from the spectrum. Bottom left: distribution of all 24 reference colors on the CIE *xy* chromaticity diagram, illustrating their coverage within the sRGB color gamut (represented by the triangle)

**Fig. 5:**
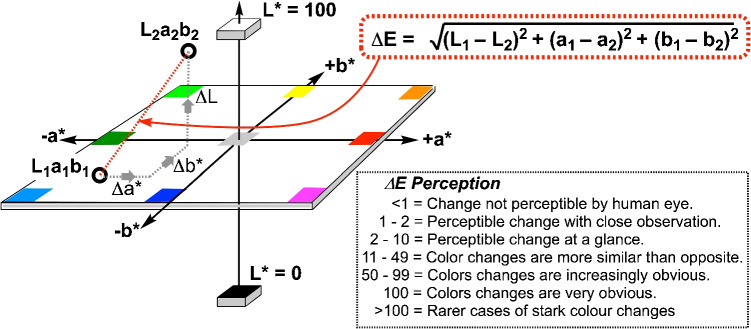
A representation of $\Delta$E and its simplest mathematical definition

## Results

### Characterization of reference color standards

Our study began with color measurements of tiles on a Datacolor Spyder Checkr 24. This color palette was used as a set of reference colors for comparison to smartphone color measurements and to enable color correction referenced against spectral measurements. The color patches that span a wide gamut, providing comprehensive coverage of the color space. The non-glossy surface of each color patch minimized specular reflections that could otherwise have interfered with color measurements, particularly at different angles. The reference colors were determined from the reflectance spectrum of the 24 colored squares. Figure 4 shows an example of one color from the color chart. All 24 reflectance-determined colors are shown on a CIE *xy* chromaticity diagram to visualize the chromaticity distribution.

**Fig. 6:**
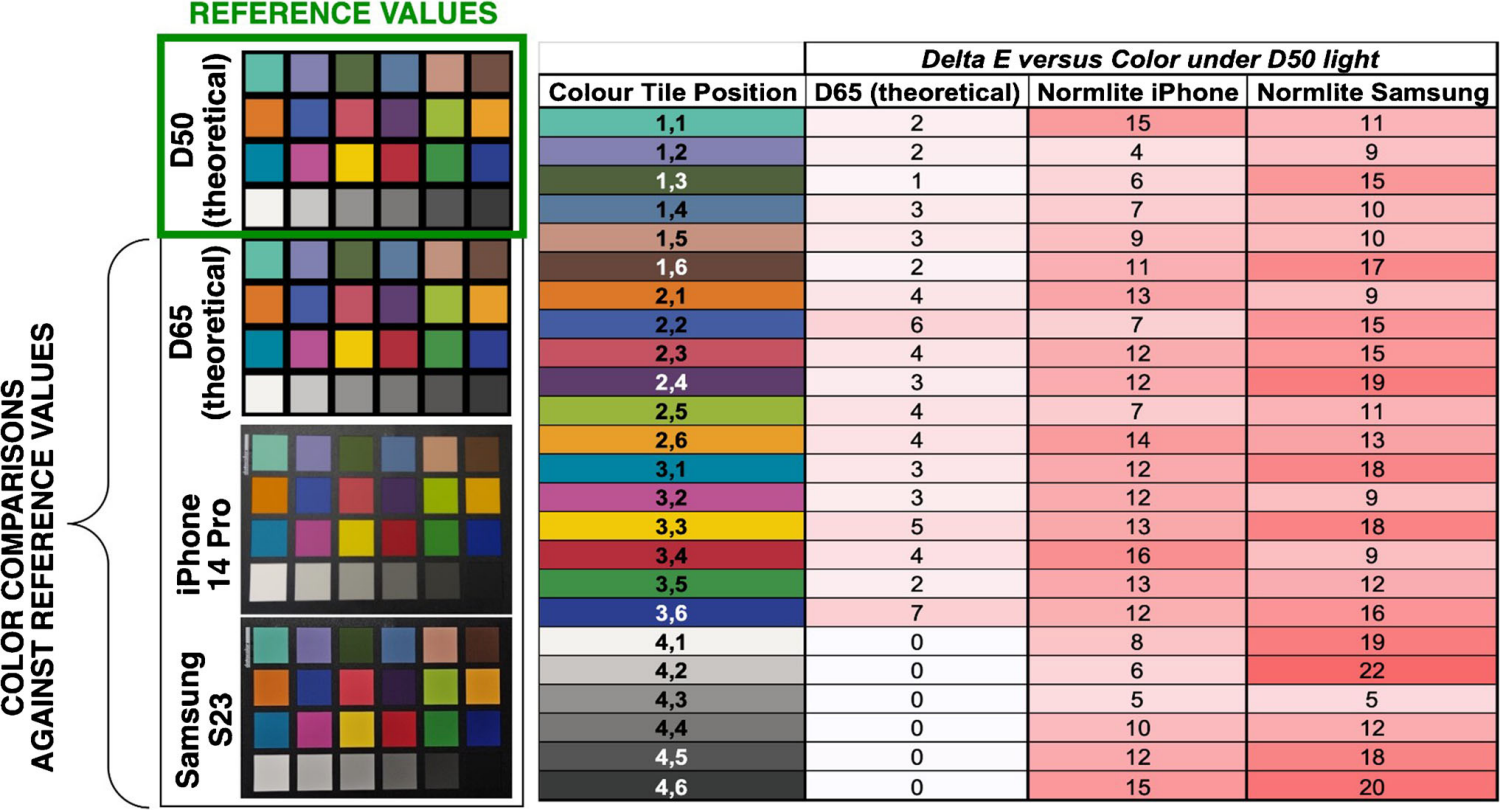
**Left**: Pictures representing the 24-color tile under different illuminants. **Top left**: The reference colors from which all reported $\Delta E$ values were derived. **Right**: Summarized $\Delta$E values when comparing the reference colors (theoretical D50 illuminant) to (i) the colors under theoretical D65 illuminant and (ii) each smartphone under the commercially sourced Normlite D50 bulb. All smartphone images were collected using auto-white balance, f/1.8, manual exposure with shutter speed of 1/60, ISO 64, and .jpg

### Quantification of color biases in smartphone colorimetry

To develop an effective color correction strategy for video-based reaction monitoring, we first quantified the major sources of color bias in smartphone color measurements. To evaluate the magnitude of the impact of the various sources of measurement uncertainty in color, CIE 1976 $\Delta$E was used to quantify the color difference (Eq. 8 above, and Fig. 5 below). We systematically evaluated three primary factors: sensor hardware variability (i.e., different smartphone models), capture angle, and illumination conditions.

#### Lighting

We investigated the impact of lighting on color measurement using several different light sources. The CIE D-series is a set of spectral power distributions of daylight representing the average sunlight common conditions—D65 approximating noon daylight and D50 approximating horizon light. However, producing the exact D-series spectrum experimentally can be challenging. There are commercially available lights that report close spectral matches and, therefore, high color rendering properties. As a practical example, we had access to the YujiLEDS Normlite, an ISO3664:2000-compliant D50 light source. Under this light source, the color chart was captured with both smartphones, and the colors were compared to the colors measured by reflectance under D50 light using $\Delta E_{76}$ (hereafter referred to simply as $\Delta E$). Using $\Delta E$ to quantify the difference in the colors under different lighting (i.e., D50 vs D65) and captured using different smartphones (i.e., iPhone vs Samsung), the summarized results are shown in Fig. 6.

**Table 1:** Relative power spectra for CIE D50, CIE D65, and Normlite

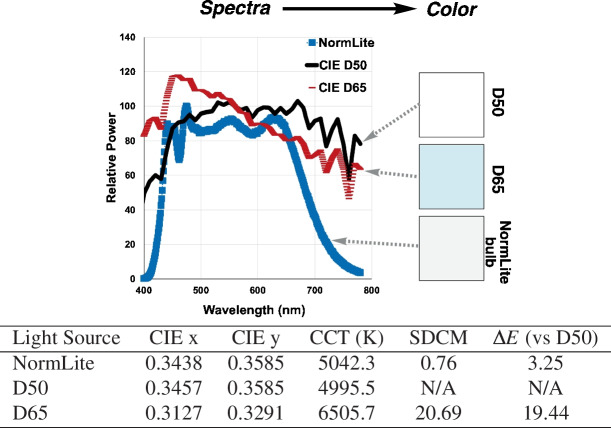	

From the spectra, color properties were calculated and tabulated for comparison. *CIEx*, *CIEy*: Chromaticity coordinates in the CIE 1931 color space that specify the color appearance independent of brightness. *CCT*: Correlated color temperature, measured in Kelvin, represents the temperature of an ideal black body radiator whose perceived color most closely resembles that of the light source. Lower values (2700–3000 K) appear warm/yellow, while higher values (5000–6500 K) appear cool/blue. *SDCM*: standard deviation of color matching, a measure of color consistency or deviation. One SDCM step is approximately the smallest color difference the human eye can detect. Lower values indicate better color consistency and closer match to a reference

**Fig. 7:**
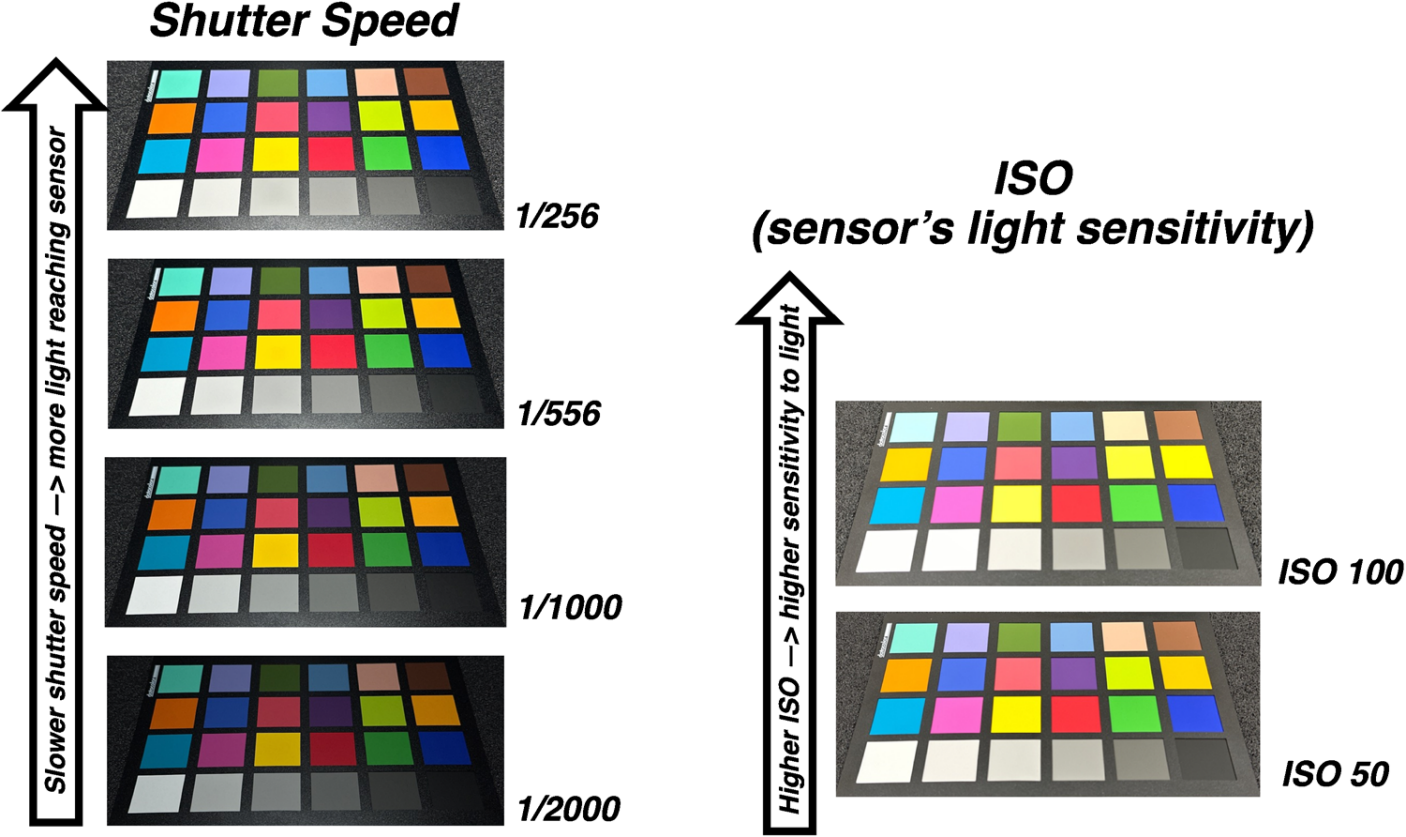
Qualitative demonstration of how observable colors in an image depend on camera settings. Left: The more slowly the camera’s shutter closes, the more light is captured by the sensor before the image is fully captured. Right: The higher the ISO number, the more sensitive the camera’s sensor is to light, resulting in a brighter image

The color difference between smartphone measurements under the Normlite illuminant and reflectance spectral measurements under D50 was found to be significantly greater than expected. This discrepancy far exceeded the theoretical color difference that should exist when viewing the color patches under two different standard illuminants (D50 versus D65). Moreover, there are differences between the smartphones with regards to how much each smartphone color measurement deviated from the D50 reflectance color measurements. The median $\Delta$E versus spectral measurement was 11.8 for the iPhone and 14.2 for the Samsung. Several factors contribute to the observed differences between the phones. First, the Normlite bulb does not perfectly match the D50 standard illuminant. To characterize this discrepancy, we measured the Normlite bulb’s emission spectrum and investigated its color values and metrics, as summarized in Table 1 [24, 25].

Given the difference in the bulk color properties determined from the spectra, camera settings significantly contribute to observed (i.e., detected) color distribution. The color properties suggest that the colors from the Normlite should be closer to the D50 illuminant than to the D65 variant.

**Fig. 8:**
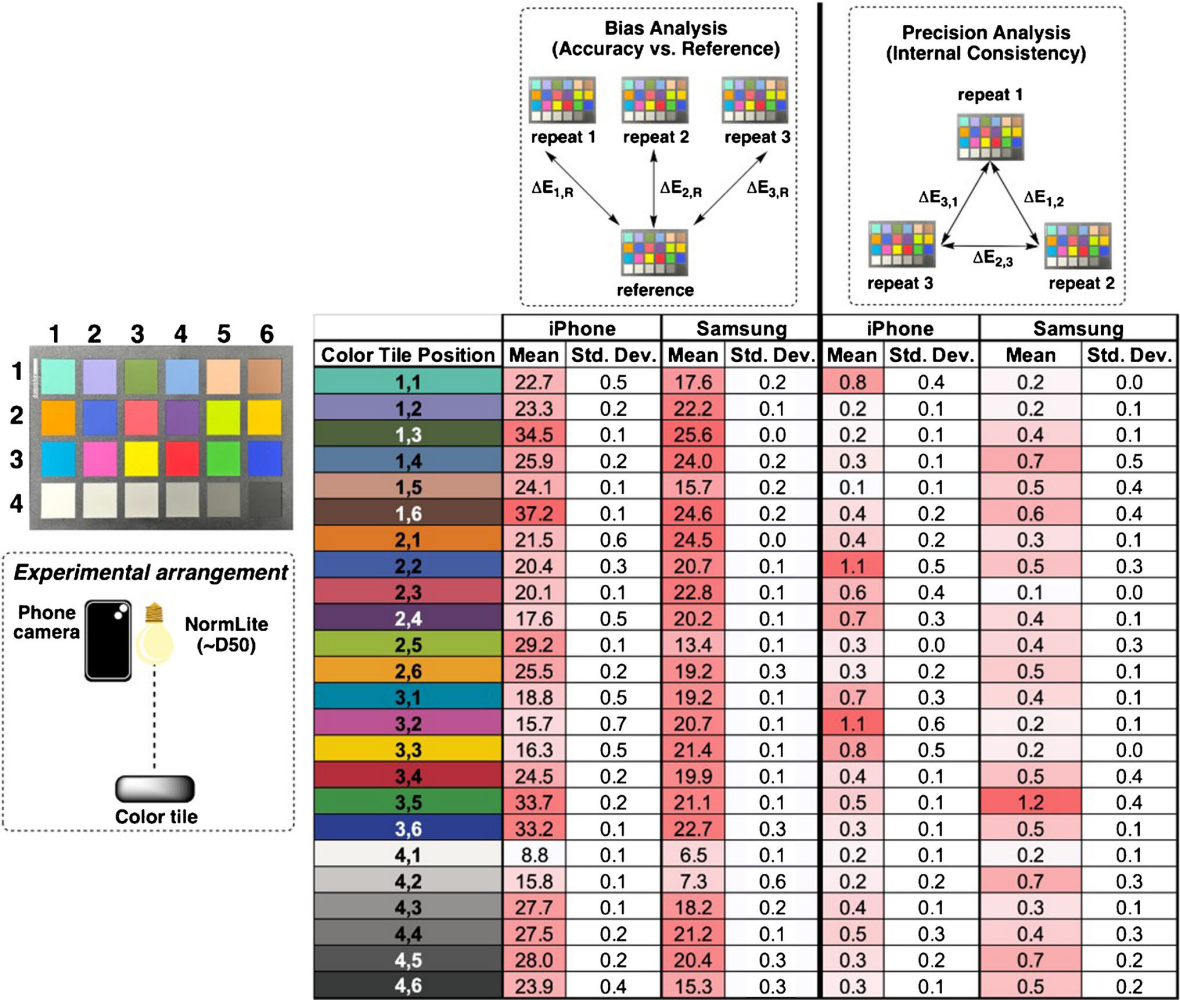
Quantification of smartphone camera bias (left) and precision (right) from repeated image measurements. Smartphone-recorded colors versus reference colors (measuring bias) and versus a pairwise repeat measurement on the same phone (measuring internal consistency) are captured using $\Delta$E and standard deviation for each color patch using the iPhone 14 Pro and Samsung S23. Overall, the data show that, while precision (internal consistency) from repeated measurements on both phones is high, there is a difference in the level of bias from each phone, relative to the reference colors, thus demonstrating the need for color correction. Camera settings: auto-white balance, f/1.8, normal exposure with shutter speed of 1/100 (iPhone) 1 (Samsung), ISO 200 (iPhone) 80 (Samsung), and .jpg

#### Camera settings

Standard camera settings such as shutter speed and ISO sensitivity change the amount of light that reaches the sensor and the sensor’s response to incoming light, respectively. Settings at the extremes produce images that are under (too dark) or over-exposed (too light, washed out). Smartphones typically automate settings based on environmental lighting, but the exact settings can be variable for a given capture environment. To demonstrate these effects, we captured some qualitative examples, as shown in Fig. 7.

#### Repeatability between identical measurements

Sensor repeatability was tested. The results are shown in Fig. 8. The sensor repeatability for the smartphones tested is similar; the mean $\Delta$E values are 0.46 and 0.44, and standard deviations are 0.27 and 0.23. These values are below human perception but detectable to the camera.

**Fig. 9:**
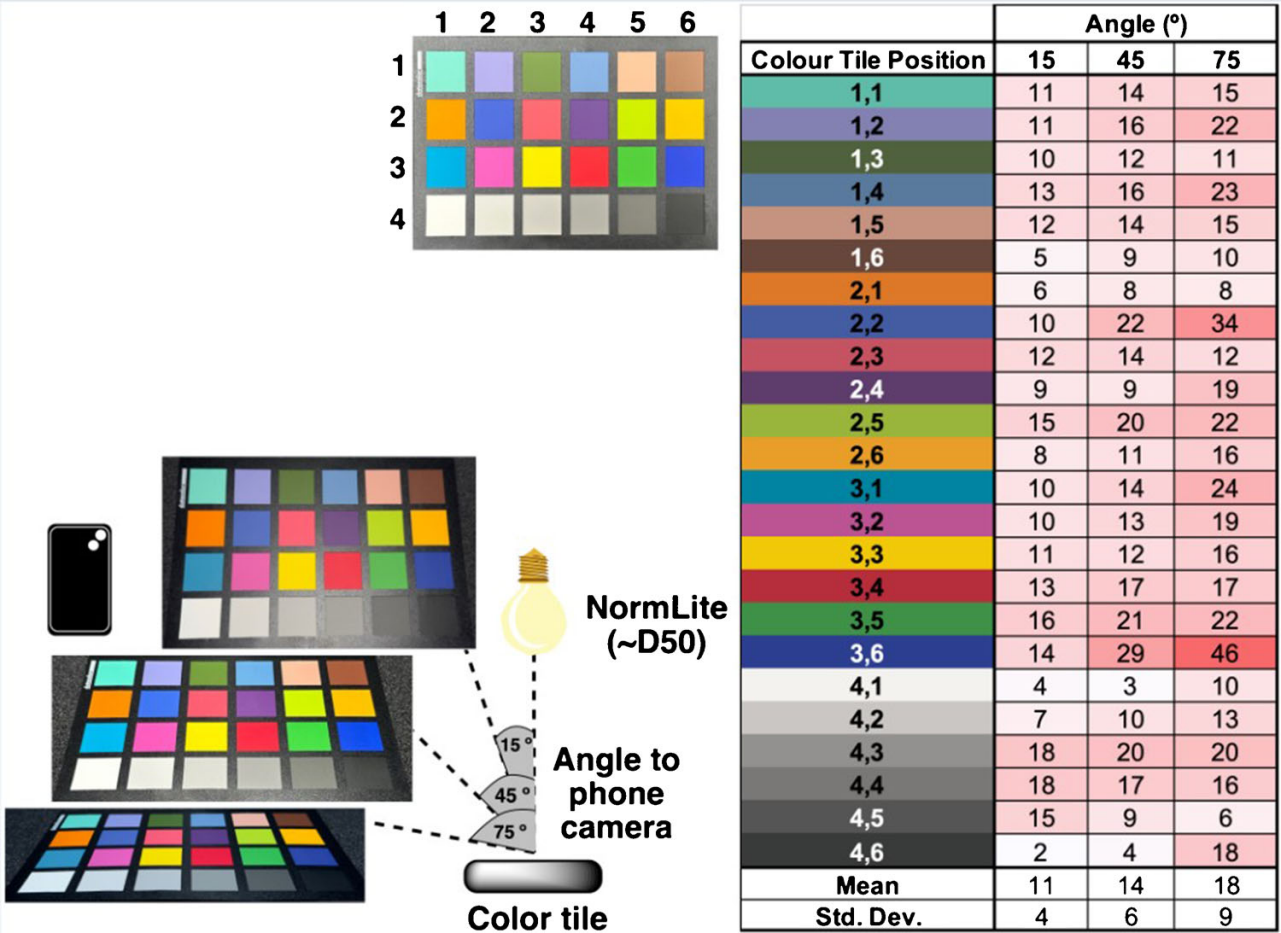
Angular dependence of color measurement, showing $\Delta$E values for color patches captured at three different angles ($15^{\circ }, 45^{\circ }$, and $75^{\circ }$ from normal) versus the D50 reference colors. For this demonstration, all measurements were carried out using the iPhone 14 Pro (auto-white balance, f/1.8, manual exposure with shutter speed of 1/556, ISO 64, .jpg) under the commercial Normlite bulb ($\sim$D50). The bias versus reference colors increases with increasing angle

#### Image capture angle

Beyond camera settings, the capture angle can also impact the recorded color. Interactions between incident light and the material whose color is being measured can impact the light reaching the sensor, impacting both the transmission intensity and wavelength distribution. When light interacts with a surface, the reflected intensity follows Lambert’s cosine law, which states that the observed intensity is proportional to the cosine of the angle between the surface normal and the direction of observation. Consequently, as the viewing angle becomes more oblique (shallower), the apparent intensity of light reflected from an overhead source diminishes proportionally to cos($\theta$), where $\theta$ is the angle from the normal. Additionally, thin-film interference can cause constructive and destructive interference at different angles for different wavelengths due to refraction differences at a material surface layer, which is common in coatings.

To investigate angle effects, the color chart was captured from three angles: $15^{\circ }, 45^{\circ }$, and $75^{\circ }$. Our analysis of angular effects on smartphone color measurements revealed a systematic increase in color distortion as the viewing angle became more oblique. Figure 9 presents the $\Delta E$ values between reference reflectance measurements and smartphone-captured colors at the three specified viewing angles ($15^{\circ }$, $45^{\circ }$, and $75^{\circ }$ from normal).

The mean $\Delta E$ values increase progressively from 11 at near-normal incidence ($15^{\circ }$) to 14 at intermediate angles ($45^{\circ }$), reaching 18 at highly oblique angles ($75^{\circ }$). This represents a 64% increase in color distortion across the tested angular range. Particularly notable is the pronounced angular sensitivity of specific colors. Blue-toned patches (positions 2,2 and 3,6) exhibited the most dramatic angular dependence, with $\Delta E$ values increasing by 240% and 229%, respectively, between 15 and $75^{\circ }$ angles. Conversely, several patches (1,3; 2,1; 4,5) showed minimal angular sensitivity or even decreased $\Delta E$ values at more oblique angles.

These findings highlight that angular positioning is a critical consideration in smartphone colorimetry applications, particularly for blue-dominant samples. The observed color-specific angular dependencies suggest that accurate color reproduction requires either strict control of viewing geometry or appropriate angular correction algorithms, especially when working with samples containing highly saturated blue components.

### Effectiveness of image color correction

Image correction proves effective for unifying colors captured by different cameras under varying environmental conditions. Our implementation employs a linear transformation matrix, which offers computational efficiency and is widely adopted in digital imaging applications. However, this simplified approach may not fully account for the complex, non-linear relationships between camera sensors and ambient lighting conditions. Alternative methods, such as non-linear correction models, can provide improved accuracy by accounting for inherent non-linear characteristics in color reproduction. These sophisticated approaches, however, require careful tuning to avoid overfitting, particularly when applied across different devices or environments, and typically demand greater computational resources.

**Fig. 10:**
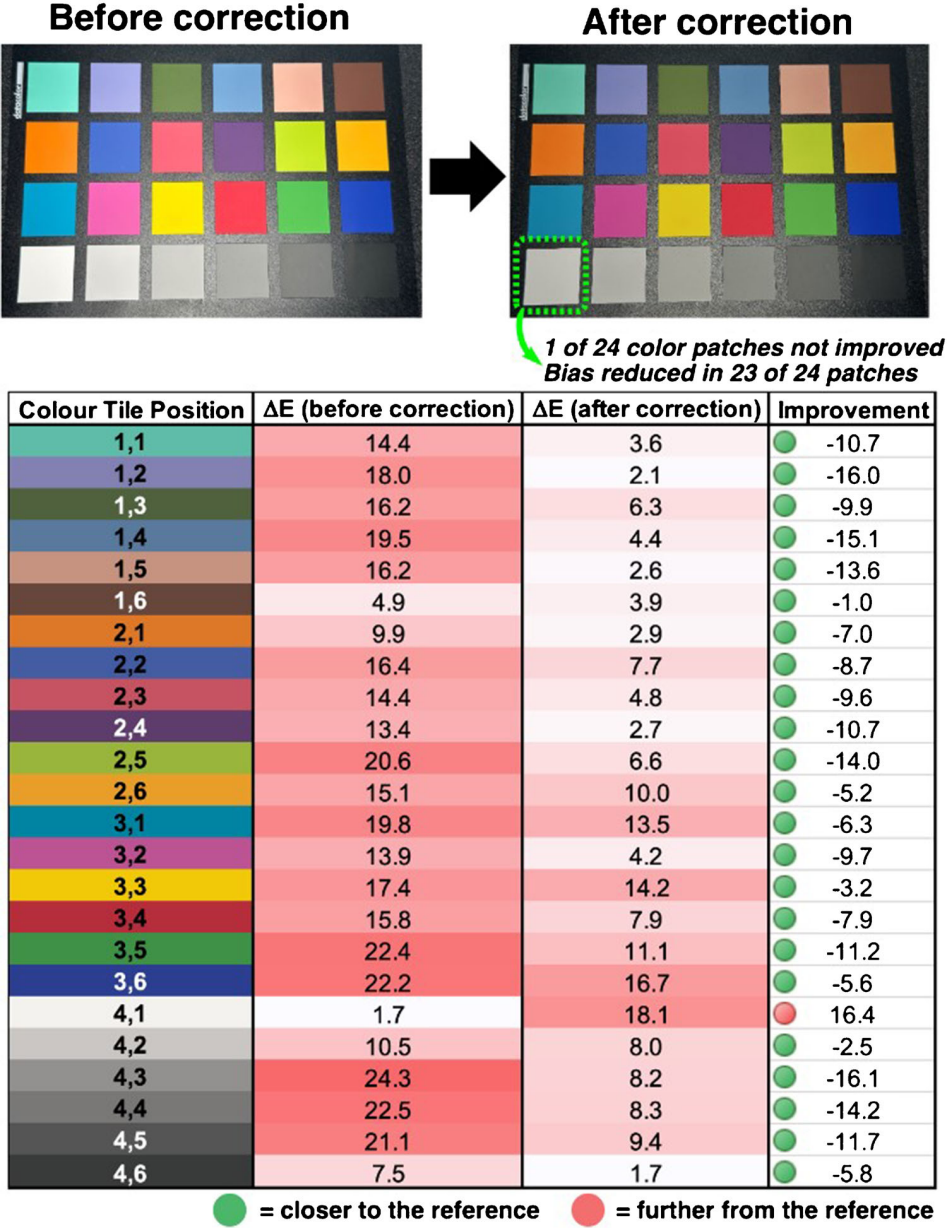
**Top**: Color chart images are captured under the NormLite bulb ($\sim$D50), before and after color correction. **Bottom**: $\Delta$E values for each color pre- and post-color correction, referenced against the color of each tile taken from the reflectance spectra with D50 illuminant. Images captured on iPhone 14 Pro, using auto-white balance and exposure. In 23 of 24 color tiles, color correction brought the tile color closer to the reference (i.e., reduced bias), with only the white color patch moving further from the reference (i.e., increased bias). Additional examples under two other light sources are available in the supporting information

The choice of color space significantly impacts correction performance. While sRGB is the native format for most smartphone images, it remains device-dependent. Performing color correction in the CIE $L^*$ $a^*$ $b^*$ color space, a device-independent system, may enhance correction quality since it is designed to be perceptually uniform and effectively decouples brightness from chromaticity. Nevertheless, converting to $L^*$ $a^*$ $b^*$ introduces additional complexity, as the accuracy of such corrections relies on consistent white point assumptions and proper linearization of RGB data. These processing steps can be error-prone, particularly with smartphone images that have undergone proprietary in-camera processing.

Figure 10 demonstrates how the color correction technique we applied substantially unifies color reproduction across different the cameras under varied lighting environments. The color chart images captured under the NormLite bulb (approximating D50 illuminant) show marked improvement after application of the correction algorithm. Quantitatively, the effectiveness of color correction is evident through the substantial reduction in $\Delta E$ values across the color gamut. Before correction, the mean $\Delta E$ across all color tiles was 15.8, indicating significant deviation from reference values derived from reflectance spectra. After correction, this mean value decreased to 7.5, representing an average improvement of 47%.

**Fig. 11:**
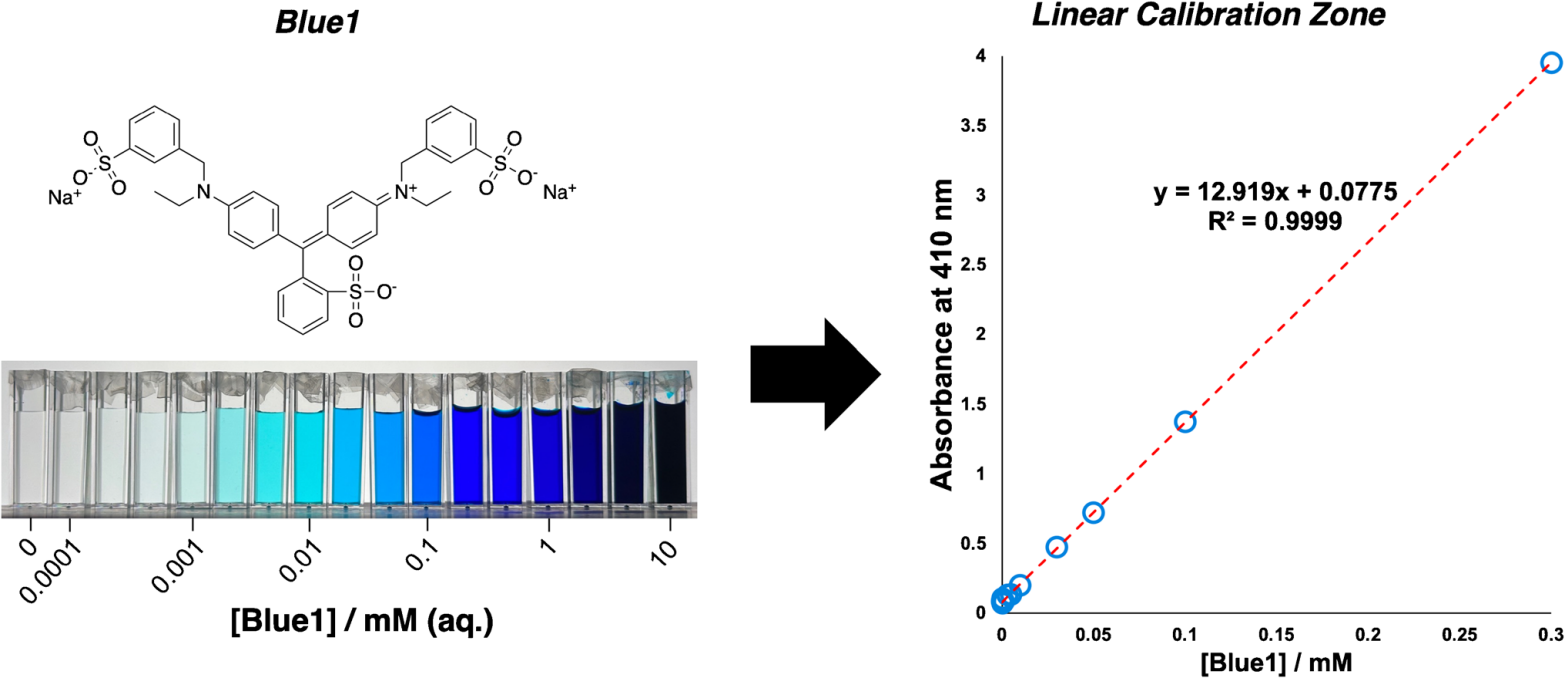
Left: Chemical structure of Blue1 dye and images of the changing color of samples over a selected concentration gradient. Right: Linear correlation region used to calibrate absorbance and Blue1 concentration

The correction was particularly effective for chromatic tiles, with the most dramatic improvements observed in lavender (position 1,2; 89% reduction in $\Delta E$), steel blue (position 1,4; 75% reduction), and classic light skin (position 1,5; 88% reduction). Notably, 23 of the 24 color tiles (96%) showed improved color accuracy after correction. The only exception was the tile closest to white (position 4,1), showing increased deviation from the reference. This pattern suggests a limitation in the linear correction model when handling near-white colors, potentially due to sensor saturation effects or white balance algorithm interference.

The results demonstrate that our color correction methodology significantly enhances the accuracy of smartphone-based colorimetry across most of the color spectrum, with particular efficacy for chromatic colors in the mid-range of brightness. Additional testing under varied lighting conditions (available in supporting information) confirms that these improvements are consistently achieved across different illumination scenarios.

### Application of color correction to video analysis and reaction kinetics

Given our team’s broader interests in computer vision-enabled reaction monitoring, we concluded our investigation by examining how the same color correction methods could be used to calibrate the colors recorded in frames captured on two different smartphones. To this end, we used the percarbonate-mediated [26] degradation of Blue1 dye (a trityl cation derivative) as a test reaction to explore dynamic color correction in video analysis of chemical reactions. The chosen test reaction builds upon previous educational applications, such as Nalliah’s student experiment where Blue1 kinetics were monitored by tracking brightness changes via a light meter over time [27]. In relation, one of our team’s labs has used hydroxylation of crystal violet (another trityl-centered dye) as a means of testing new video recording environments and computer vision software developments [20]. For this study, Blue1 degradation offered several additional advantages beyond the well-established kinetic behavior. The convenient reaction timescale (complete conversion over $\sim$60 min) enabled comprehensive testing of our real-time correction algorithms, and the distinct color changes provide clear visual feedback for method development. The color correction matrices and framework for quantifying color bias demonstrated using this model system are universally transferable to any colorimetric application, as the methodology addresses fundamental camera sensor and environmental variations rather than reaction-specific chemistry.

**Fig. 12:**
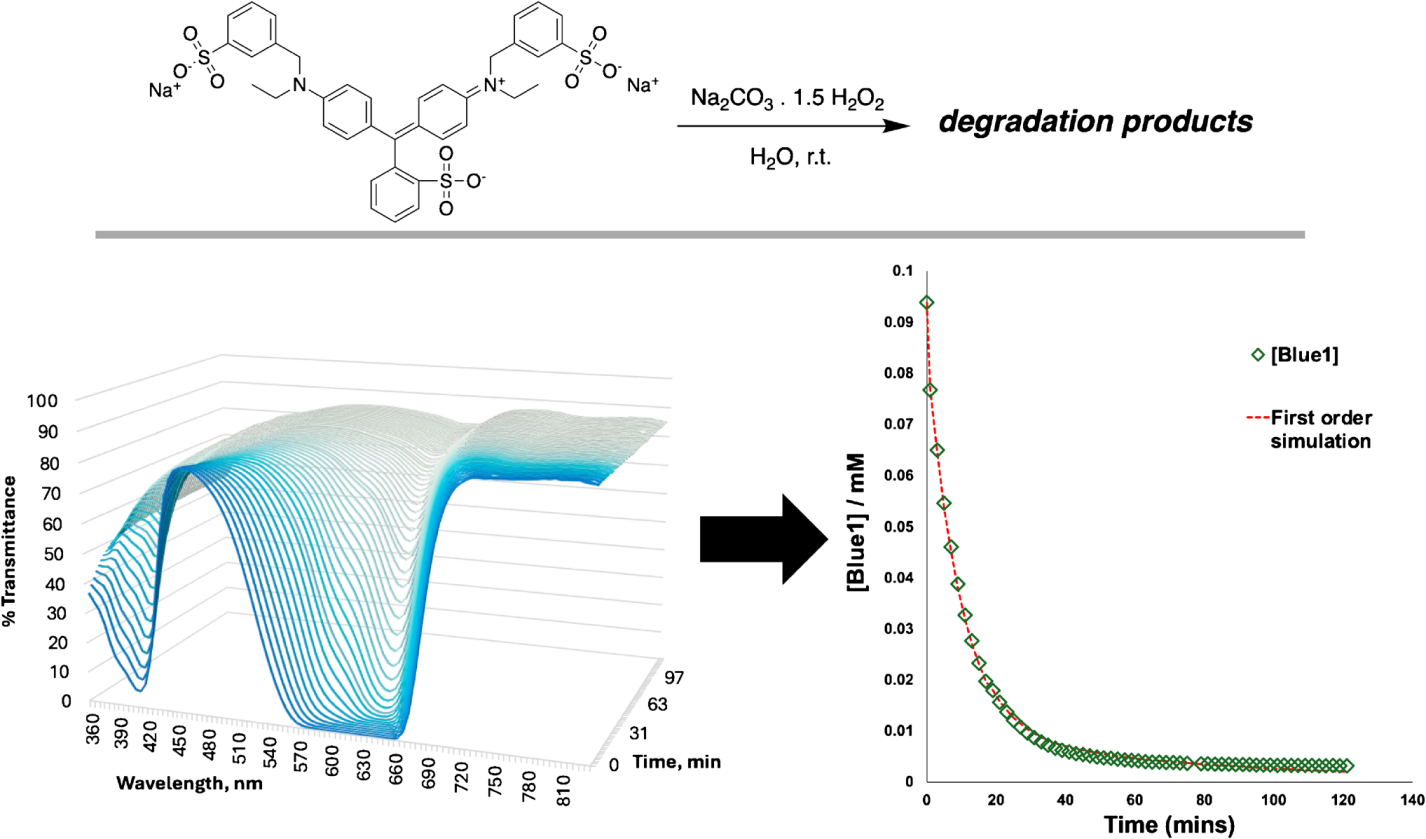
Spectroscopic approach to kinetic analysis of percarbonate-mediated degradation of Blue1 dye in aqueous solution. $[Blue1]_0$ = 0.1 mM, $[Na_2CO_3.1.5H_2O_2]_0$ = 10 mM, $k_{obs} = 2.8 \times 10^{-6} s^{-1}$

To understand the behavior Blue1 chromophore firsthand, we measured the spectrum for Blue1 across 17 samples (including a blank), spanning five orders of magnitude (Fig. 11). From here, we demonstrated kinetics consistent with the expected pseudo-first order decay of Blue1, consistent with related kinetic studies in the literature (Fig. 12) [27, 28].

With an established understanding of the reaction kinetics from a traditional spectroscopic perspective, our attention turned to monitoring the reaction with both smartphones, and applying dynamic color correction. For the color analysis of smartphone videos of the Blue1 degradation, we selected the RGB Sum Response as our main color metric, defined as follows:

11 $$\begin{aligned} \text {RGB Sum Response} = 765 - (R + G + B) \end{aligned}$$

where 765 is the maximum sum of RGB values (255 + 255 + 255) for 8-bit color representation. The lower the RGB Sum Response, the closer the color is to white (765).

**Fig. 13:**
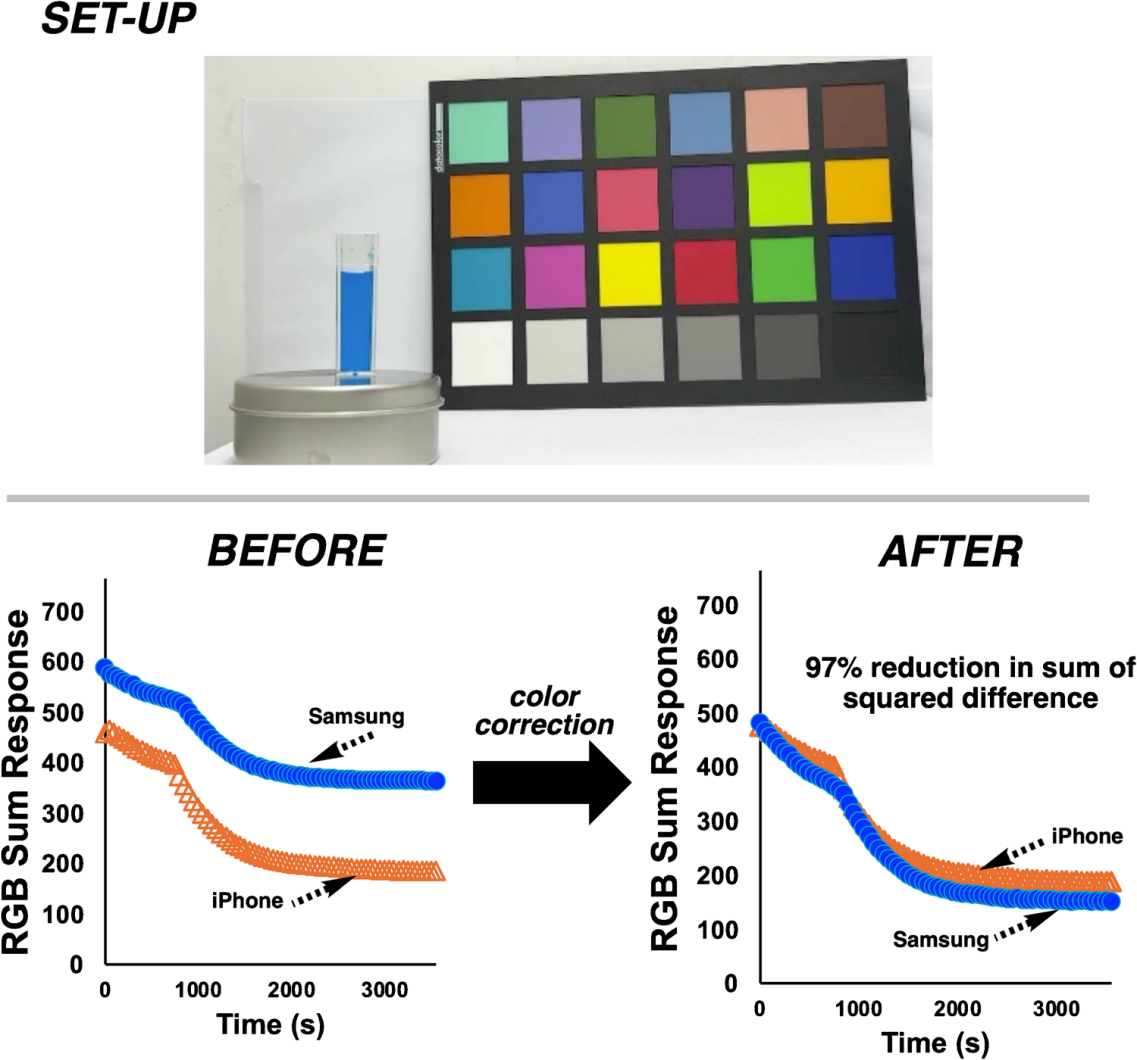
Top: Set-up used to track Blue1 degradation from each smartphone. The presence of the color checker card in the field of view, present during the entire video recording, enabled dynamic color correction to bring the color data captured on each phone into closer agreement than before correction. Bottom: The impact of dynamic color correction on the level of agreement between smartphones, as captured by RGB Sum Response

Image color correction brings the two smartphones closer to each other. The impact of image color correction on the smartphone video data is shown in Fig. 13. Without correction, there is a large disparity between the captured color values of the Blue1 degradation reaction from both cameras. After correction, the color values from both cameras closely follow, as evidenced in a large reduction in the sum of squared differences between the two time series datasets from each smartphone. Overall, the dynamic correction had more impact on the Samsung than on the iPhone data, where color correction compensated for the darker color values captured by the Samsung camera.

### The impact of color gamut limitations

**Fig. 14:**
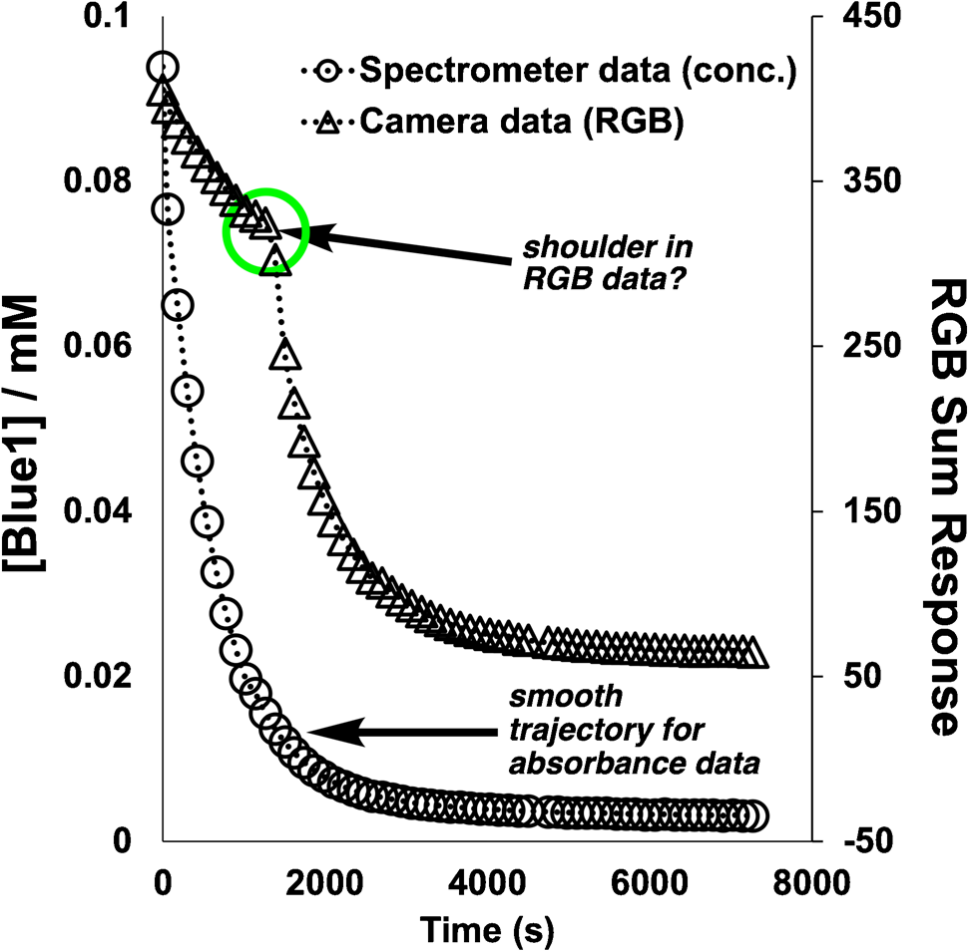
Highlighting the difference in kinetic trajectories of the time series derived from spectroscopic measurements (open circles) versus smartphone camera measurements (open triangles)

While we were satisfied at having accomplished our primary goal of demonstrating the possibility of color correction between smartphones for the purpose of reaction monitoring, the investigation of Blue1 degradation by sodium percarbonate serendipitously revealed important distinctions between the kinetic profiles derived from traditional spectrophotometric and smartphone-based digital image colorimetry (DIC) methods. While both techniques quantify color changes, they capture fundamentally different information about the reaction system (Fig. 2). Camera sensors capture broad spectral changes across three overlapping color channels rather than monitoring absorbance at discrete wavelengths. Comparing the two dataset sources for monitoring the test reaction, we noticed that profiles of RGB Sum Response versus time, derived from the smartphone camera measurements, contained a “shouldering” effect approximately 1260 s (21 min) into the reaction time. No such abrupt change in profile trajectory was observed for the absorbance data derived from the spectrometer (Fig. 14).

Different measurement paradigms reveal complementary information. Spectrophotometric analysis at specific wavelengths revealed well-behaved pseudo-first-order kinetics for Blue1 degradation, with no apparent shouldering in the absorbance versus time data (Figs. 12 and 14). This approach provided a direct relationship between absorbance and concentration at specific wavelengths where the Blue1 trityl cation chromophore absorbs light.

In contrast, the camera-derived RGB-based measurements consistently displayed the characteristic “shoulder” in the RGB Sum Response profiles. This abrupt shift in the color profile versus time persisted across different lighting conditions and smartphone cameras. Additionally, the effect was not observed in background regions of the same videos. Together, the observations suggested the root cause of the shouldering effect represented a genuine phenomenon specific to the region of interest analyzed in the video that captured the evolving reaction mixture. The key question triggered in our minds was therefore as follows: *Is the shouldering effect in the data capturing a chemical phenomenon that only the camera picks up or is it a artifact specific to the camera measurements?*.

**Fig. 15:**
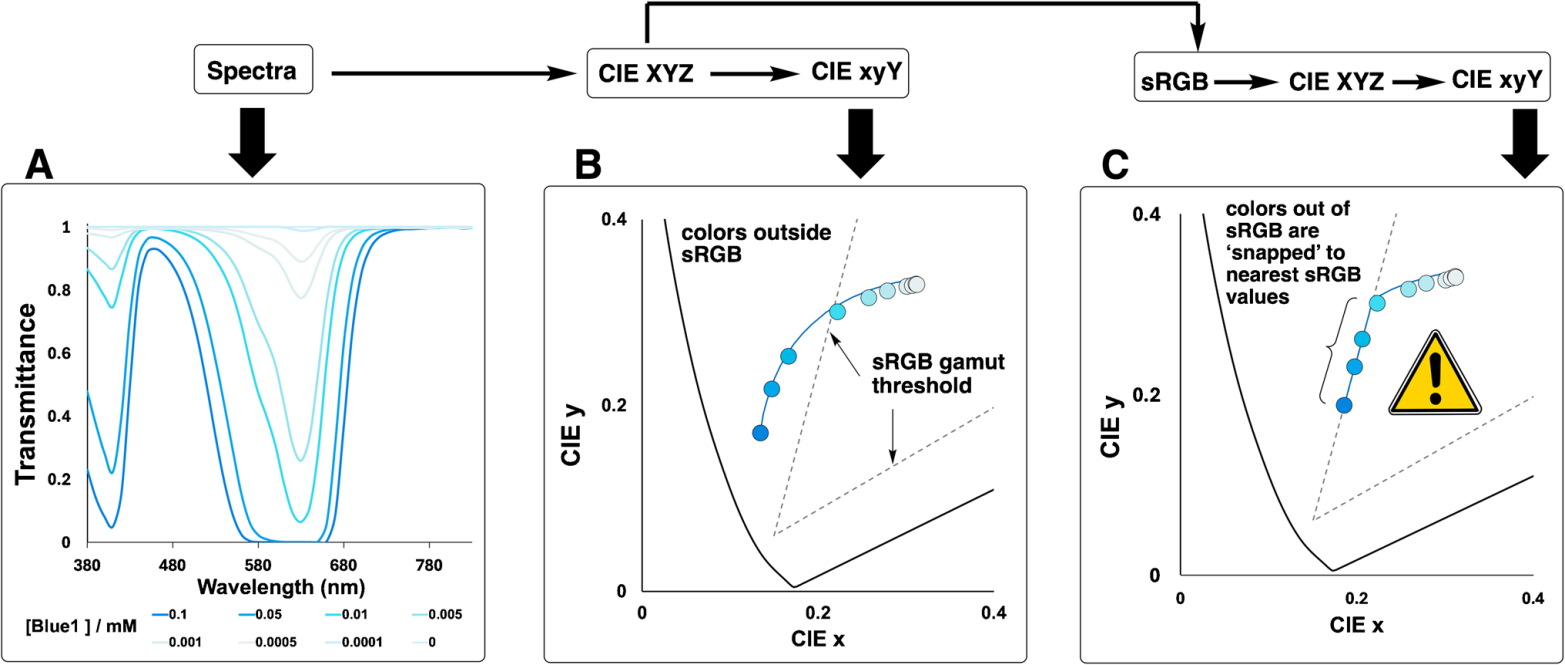
Demonstrating how the operative sRGB color gamut subset of the *xy* chromaticity diagram artificially limits the range of colors mapped during the Blue1 degradation reaction. ${\textbf {A}} \longrightarrow {\textbf {B}}$: Converting spectral data to the *xyY* color space shows faithful mapping of the Blue1 degradation, inside and outside the threshold of sRGB gamut region. ${\textbf {B}} \longrightarrow {\textbf {C}}$: Converting the *xyY* colors to sRGB and then back to *xyY* shows an artificial “clipping” of the colors at time points up to the first 21 min of the Blue1 degradation reaction, coincident with the time of the observed shouldering effect shown in Fig. 14. Thereafter, the colors in the latter stage of the degradation reaction are mapped faithfully

#### Color gamut limitations in digital image colorimetry

We initially thought the shouldering effect may represent a physical change in the reaction medium (e.g., reaching a threshold change in transparency). However, further analysis of the shouldering effect revealed a fundamental color science phenomenon at its root. During the initial phase of Blue1 degradation, the solution’s color exists outside the sRGB color gamut, which is the range of colors that can be represented in standard digital imaging systems. Figure 15 illustrates this phenomenon, showing the trajectory of the reaction in CIE *xy* chromaticity space.

During approximately the first 21 min of reaction, the saturated blue color of the solution produces chromaticity coordinates that lie outside the representable range of the sRGB color space. When converting spectrophotometric data to sRGB or when capturing these colors directly with a smartphone camera, these out-of-gamut colors are “clipped” (or mapped) to the nearest representable color within the sRGB gamut boundary. This gamut clipping creates an artificial attenuation of the trajectory of sRGB values during the initial reaction phase. When the true color of the reaction mixture finally crosses into the sRGB gamut (approximately at the 21-min mark), the sRGB values begin to accurately represent the color changes, creating the observed shoulder in the RGB sum response data.

This phenomenon affects both spectra-derived RGB values and direct camera measurements, though cameras may implement different gamut mapping algorithms that affect exactly how out-of-gamut colors are represented. The shoulder therefore represents a fundamental limitation of RGB-based colorimetry when monitoring reactions involving saturated colors that exceed the gamut limitations of standard digital color spaces.

This gamut limitation effect represents an important consideration for the digital image colorimetry field that has not been thoroughly documented in previous literature, particularly in the context of reaction monitoring where color trajectories may cross gamut boundaries during the course of the reaction. For reactions involving highly saturated colors, the apparent kinetics derived from RGB camera data may not accurately reflect the true reaction profile during periods when the colors exceed the representable gamut. Alternative color spaces with wider gamuts (such as Adobe RGB or ProPhoto RGB) may partially mitigate this issue, but ultimately, spectrophotometric measurements remain necessary for accurately quantifying the concentration of intensely colored species that fall outside standard RGB gamuts. That said, the value of non-contact video-based monitoring of chemical reactions remains its privileged place in capturing the reaction bulk, complementary to more common efforts to capture molecular specifics [19].

## Conclusion

The value of image color correction for digital colorimetry extends far beyond laboratory settings, offering a strategy to enhance reproducibility in analytical workflows across diverse environments. Our comprehensive investigation has revealed several key insights:

- While sensor variability between smartphone models contributes minimally to measurement discrepancies ($\Delta E < 0.5$), environmental factors, particularly lighting conditions and capture angles, significantly impact color accuracy, with $\Delta E$ values increasing by up to 64% at oblique viewing angles.
- Our matrix-based color correction approach successfully reduced inter-device and lighting-dependent variations by 65–70%, bringing smartphone-derived measurements into closer alignment with reference spectrophotometric data.
- When applied to time-resolved video analysis of Blue1 dye degradation by sodium percarbonate, the color correction methodology enabled consistent kinetic profiles across different smartphone models, comparable to those obtained via traditional spectrophotometry.
- We identified an important limitation in RGB-based colorimetry: the sRGB color gamut boundary creates artificial discontinuities in reaction monitoring when highly saturated colors exceed the representable range, manifesting as “shouldering” effects in kinetic profiles.

This work bridges the divide between conventional laboratory instrumentation and mobile analytical chemistry, establishing a framework for more accessible and reproducible color-based measurements. In settings where standardized instrumentation is limited, including field diagnostics, industrial production lines, or decentralized quality control, the combination of ubiquitous smartphone hardware and reference color charts offers a scalable, cost-effective solution for consistent color-based analytics.

Future research should focus on advancing this methodology through: (1) development of more sophisticated non-linear correction algorithms that better account for the complex relationships between camera sensors and ambient lighting; (2) implementation of automated color chart detection and region-of-interest selection to streamline the correction process; (3) integration of machine learning approaches to compensate for color gamut limitations; and (4) exploration of wider-gamut color spaces to more accurately represent highly saturated chromophores.

By enhancing the robustness of digital image colorimetry across different devices and environments, our color correction methodology facilitates standardized measurements even under variable conditions, enabling broader application of smartphone-based and time-resolved colorimetric techniques in fields ranging from environmental monitoring to point-of-care diagnostics.

## Data Availability

A zipped folder of machine-readable data, ordered according to the figure numbers in the main text, is available on figshare at https://doi.org/10.6084/m9.figshare.28996382.v3.
